# Postprandial Blood Glucose can be less than Fasting Blood Glucose and this is not a Laboratory Error

**DOI:** 10.31729/jnma.3985

**Published:** 2019-02-28

**Authors:** Vivek Pant, Keyoor Gautam, Santosh Pradhan

**Affiliations:** 1 Samyak Diagnostic Pvt. Ltd, Lalitpur, Nepal

**Keywords:** *clinical laboratory*, *diabetes*, *fasting blood glucose*, *postprandial blood glucose*

## Abstract

Higher fasting blood glucose level than postprandial level can be seen in variety of conditions in both normal population and diabetics. Various modifiable factors along with underlying condition of patient behind such laboratory picture are discussed in this article.

## INTRODUCTION

In clinical laboratories, daily numerous blood samples are processed for estimation of fasting blood glucose (FBG) level and postprandial blood glucose (PPBG) level. It is found in some cases that postprandial level of blood glucose is remarkably lower than that of fasting level.

Being unsatisfied with the laboratory result, patient or sometimes clinician wants to recheck the blood glucose level in next laboratory. This is because of common perception that PPBG level must be higher than FBG level. But the repeated investigation yields similar type of result. FBG may be higher than the PPBG in both diabetics and healthy population. Many modifiable factors may be the cause for this laboratory finding along with underlying conditions of such patient.

The aim of the present article is to aware healthcare providers about the factors that can cause FBG level higher than PPBG.

## DISCUSSION

Blood glucose level primarily depends upon individual characters like type and quantity of food intake, physical activity and the body's metabolic response. Pre-analytical factors that affects blood glucose level are smoking, caffeinated drinks, use of hypoglycemic drugs, heavy exercise, anxiety and delay in sample processing.^1,2^ Careful attentions to these modifiable factors by both clinician and laboratory staff are essential to ensure accurate glucose measurement.

The organ that is responsible for fasting value of blood glucose is liver whereas it is the pancreas that is responsible for PPBG value. Several hours after dinner, blood glucose level drops leading to decrease in insulin level and rise in glucagon level. Glucagon is responsible for maintaining adequate blood glucose level in fasting condition via activation of metabolic pathways like gluconeogenesis and glycogenolysis in liver. Higher FBG level is due to increase in glucagon to insulin ratio as seen in diabetes, where liver is involved in excess glycogen breakdown and gluconeogenesis. Despite having sufficient insulin, individual may have higher FBG value which is mainly due to insulin resistance that is the commonest cause of impaired fasting glucose tolerance and diabetes mellitus.

Apart from this underlying disturbed homeostatic response of person, various modifiable factors have effect on FBG level. Higher FBG is mainly due to high carbohydrate meal at bedtime or not enough diabetic medication. The anxious individual with disturbed sleep may also have high FBG. Furthermore, the lesser known entities like Dawn phenomenon and Somogyi effect also contribute to higher FBG in the morning. These are the body's response to hypoglycemia by release of counter regulatory hormones like glucagon, epinephrine and cortisol which can be treated by altering type and time of meal and medication.

Immediately after food intake, insulin is released from pancreas that maintains blood glucose by activating glycolysis pathway along with suppression of glycogenolysis and gluconeogenesis pathway. The common cause of decreased postprandial glucose level is intake of antidiabetic medication and strenuous activity before sampling. Individuals with lesser PPBG level than FBG should be evaluated for possibility of meal induced hypoglycemia also known as reactive hypoglycemia. Various causes for this are high insulin sensitivity, exaggerated response of glucagon-like peptide-1, defects in counter regulatory hormones like glucagon and massive weight reduction.^36^ Chewing and eating slower can reduce the reactive glucose surge post meal. Other causes of lower PPBG includes gastroparesis seen in diabetic patient which an alter the rate and amount of food passing into small intestine causing erratic changes in blood sugar level. Some people deliberately eat less or eat non-carbohydrate meal before testing for PPBG level.

It should be noted that fasting and postprandial glucose level help us adjust the dose of diabetic medications properly and glycated hemoglobin (HbA1c) helps to know whether there is an overall control so as to prevent complications. Due to individual variation of FBG and PPBG and large imprecision in analysis, some researchers have advocated the use of HbA1c only for diabetes diagnosis.^7^

It is essential to understand that laboratory error, though present in few cases is not always the cause for higher fasting blood glucose level than postprandial level. Healthy subjects with such laboratory finding should be followed for possibility of getting diabetes mellitus type 2 and should be advocated for lifestyle change along with dietary modification. In diabetics, changing the medication dosage, form or time should be considered along with counseling for aforementioned factor that affects laboratory result.
